# Contribution of anaerobic energy expenditure to whole body thermogenesis

**DOI:** 10.1186/1743-7075-2-14

**Published:** 2005-06-15

**Authors:** Christopher B Scott

**Affiliations:** 1 Department of Sports Medicine, University of Southern Maine, 37 College Avenue, Gorham, ME 04038, USA

## Abstract

Heat production serves as the standard measurement for the determination of energy expenditure and efficiency in animals. Estimations of metabolic heat production have traditionally focused on gas exchange (oxygen uptake and carbon dioxide production) although direct heat measurements may include an anaerobic component particularly when carbohydrate is oxidized. Stoichiometric interpretations of the ratio of carbon dioxide production to oxygen uptake suggest that both anaerobic and aerobic heat production and, by inference, all energy expenditure – can be accounted for with a measurement of oxygen uptake as 21.1 kJ per liter of oxygen. This manuscript incorporates contemporary bioenergetic interpretations of anaerobic and aerobic ATP turnover to promote the independence of these disparate types of metabolic energy transfer: each has different reactants and products, uses dissimilar enzymes, involves different types of biochemical reactions, takes place in separate cellular compartments, exploits different types of gradients and ultimately each operates with distinct efficiency. The 21.1 kJ per liter of oxygen for carbohydrate oxidation includes a small anaerobic heat component as part of anaerobic energy transfer. Faster rates of ATP turnover that exceed mitochondrial respiration and that are supported by rapid glycolytic phosphorylation with lactate production result in heat production that is *independent *of oxygen uptake. Simultaneous direct and indirect calorimetry has revealed that this anaerobic heat does not disappear when lactate is later oxidized and so oxygen uptake does not adequately measure anaerobic efficiency or energy expenditure (as was suggested by the "oxygen debt" hypothesis). An estimate of anaerobic energy transfer supplements the measurement of oxygen uptake and may improve the interpretation of whole-body energy expenditure.

## Background

"...(animals) *take up oxygen and complex compounds made by plants, discharge these compounds largely in the form of carbonic acid *(CO_2_)*and water as the products of combustion and partly as simpler reduced products, thus consuming a certain quantity of chemical potential energy, and generate thereby heat and mechanical energy*" (H.L.F. Helmholtz, 1821-1894)

Measurements of heat loss and oxygen uptake are the two major methods for determining energy expenditure although they do not always provide equivalent results at equivalent time points [1-4]. The focus on oxygen uptake follows from the extensive involvement of mitochondria in ATP re-synthesis accompanied by concomitant heat production [5-8]. Sites of ATP hydrolysis (e.g. contracting muscle) represent another source of energy transfer and heat exchange. Non-steady state periods of rapid growth and development, disease, arousal from torpor, heavy/severe exercise and hypoxia, however, offer proof of how tenuous the relationship between heat loss and oxygen uptake can be [1,3,4,9-11]. In isolated mammalian cells, for example, the accelerated production of lactate has been shown to make a substantial contribution to heat production beyond mitochondrial (aerobic) involvement [12]. If heat serves as the standard measure of energy expenditure then anaerobic energy transfer, specifically rapid glycolysis and glycogenolysis with lactate production (i.e., rapid anaerobic ATP re-synthesis) has the potential to make significant contributions to cellular energy expenditure.

Glycolysis as a form of fermentation has been a part of life for an estimated three billion years [13]. It has been observed that anaerobic glycolysis and oxygen uptake often behave in a reciprocal manner. Pasteur, for example, demonstrated that glucose utilization in yeast was more rapid when oxygen was absent [14]. It was subsequently hypothesized that alterations in aerobic respiration influence glycolytic rate. Crabtree [15] described the suppression of oxygen uptake when an abundance of glucose was provided to tumor cells. More recently it has been shown that this "'Crabtree Effect" is not the result of altered respiratory function, but rather an induction of the glycolytic enzymes during cellular proliferation as lactate dehydrogenase (LDH) increased 10-fold and appeared to influence the subsequent routing of NAD^+ ^to the cytoplasm and away from immediate mitochondrial respiration [16]. As it pertains to cellular metabolism then, a distinct trade-off between anaerobic and aerobic metabolic pathways can be seen; high rates of mitochondrial ATP re-synthesis have the potential to suppress anaerobic glycolysis and, conversely, rapid glycolytic ATP re-synthesis can suppress aerobic respiration. In an experiment with yeast, the relative contributions of anaerobic and aerobic processes to total ATP re-synthesis were genetically modified by increasing the glycolytic enzyme, phosphofructokinase (PFK). This modification resulted in yeast with enhanced anaerobic ATP re-synthesis – accompanied by a 36% lower oxygen uptake – but unchanged total ATP turnover compared to normal aerobic-respiring yeast [17]. It appears then, at least in single cell-types, anaerobic ATP re-synthesis has the potential to promote a discrepancy between energy expenditure (heat loss) and oxygen uptake. The question that remains is whether similar discrepancies are seen at the level of the whole-animal.

This review contains four sections. The first briefly describes thermodynamic and bioenergetics interpretations of energy transfer. The second section describes the traditional (stoichiometry and gas exchange) and contemporary (bioenergetic) interpretations behind metabolic heat production. The third section describes energy transfer as lactate production and lactate removal. In the fourth section examples are provided that suggest how an estimate of anaerobic energy transfer along with a separate measure of oxygen uptake may better influence the interpretation of whole-body efficiency and energy expenditure.

## Energy transfer

The first law of thermodynamics states that energy can not be created or destroyed but can and does change form. The second law describes how energy is transferred from one form to another. For example heat, as an expression of energy, *always *flows in one direction – from hot to cold. Other ways of stating this are that energy flows "downhill" or, from a state of lower entropy to one of higher entropy. Entropy represents energy that is not available to perform work so that simply put, energy transfer is inefficient. Inefficiency also appears in the form of heat production that is usually discarded into the environment. In the late 1800's Josiah Gibbs acknowledged the importance of entropy and enthalpy in his explanations of chemical energy transfer. The Gibbs free energy is recognized as energy that is available to perform work at constant temperature and pressure and is the usual thermodynamic parameter for identifying spontaneity of chemical reactions. Thermodynamics was further developed in the context of a closed system where heat but not matter was exchanged with the environment (e.g. test tube reactions). This description was also applied to living cells. It is of interest that A.V. Hill shared the 1922 Nobel Prize in part for his recognition that muscle cells were not heat-to-mechanical motion converters as modeled by the steam engine, but could rather be understood as chemo-mechanical converters.

Biological energy transfer or bioenergetics is accurately described in the context of an open system where matter and energy are continuously exchanged between a cell and its immediate environment [18-20]. In open or in closed systems the Gibbs free energy 'drives' biochemical and chemical reactions, respectively. Closed systems have specific starting and ending points for the Gibbs free energy change during energy transfer. In an open system however, the Gibbs free energy availability may change as the rate of energy transfer and the ratio of product to reactant varies during the exchange (e.g., as the distance from equilibrium is altered) [2]. Within cells, heat and entropy production are the continuous result of energy transfer during ATP hydrolysis and re-synthesis, collectively known as ATP turnover. ATP undergoes hydrolysis to "fuel" a variety of cellular functions such as muscle contraction, the sodium-potassium pump within cell membranes and coupling to endergonic reactions. Aerobic and anaerobic metabolisms serve to re-synthesize ATP. Entropy production can not be directly measured. Heat loss can be quantified with direct calorimetry as a measure of energy expenditure (transient heat storage is not described here so that heat loss is equated with heat production). Heat production also can be *estimated *with measures of oxygen uptake and carbon dioxide as indirect calorimetry.

## Gas exchange and energy expenditure

Lavoisier first described both biological respiration and combustion in terms of their equivalence of gas exchange and heat production. At the end of the nineteenth century experiments by Eduard Pfluger and others compared direct measurements of heat production with indirect measures of gas exchange. Pfluger utilized a stoichiometric analysis to uncover the relationship between the chemical compositions of different foodstuff and their oxidation. From this data, for example, glucose oxidation is described as,

C_6_H_12_O_6 _+ 6 O_2 _→ 6 CO_2 _+ 6 H_2_O

The stoichiometric ratio of CO_2_: O_2 _as measured from the mouth became known as the respiratory exchange ratio (RER) and serves as a valuable means of interpreting substrate utilization and heat production. When an all-carbohydrate diet is being oxidized the RER is 1.00 (6 CO_2_: 6 O_2_) and heat production is estimated at 21.1 kJ per liter of oxygen (1 l O_2 _= 21.1 kJ). When fatty acids are the principle substrate oxidized, the RER is 0.70 (palm oil oxidation = 16 CO_2_: 23 O_2_) and one liter of oxygen uptake estimates heat production at 19.6 kilojoules (1 l O_2 _= 19.6 kJ).

The higher RER for carbohydrate oxidation has been interpreted to mean that fat oxidation requires more oxygen and results in less heat production than carbohydrate oxidation [21]; this does not signify that carbohydrate is the more efficient fuel source. Only 2-carbon intermediates (acetyl CoA) can enter into the Krebs cycle for complete aerobic oxidation and the product of anaerobic carbohydrate breakdown – pyruvate – must undergo de-carboxylation (i.e., carbon dioxide production) by the enzyme pyruvate dehydrogenase (PDH) before it can be oxidized aerobically. In comparison, fat is broken down by mitochondrial beta-oxidation enzymes into 2-carbon intermediates; no de-carboxylation takes place prior to entrance into the Krebs cycle. Per volume of ATP re-synthesized aerobically then, the complete oxidation of glucose and glycogen has additional relative carbon dioxide production, not less relative oxygen uptake, as compared to fat oxidation. The conversion of one liter of carbon dioxide into an estimate of heat production for glucose and fat oxidation reveals larger discrepancy in energy expenditure at 21.1 and 27.6 kJ, respectively [22]. If oxygen uptake better represents energy expenditure than carbon dioxide production, then it must be concluded that the ratio of CO_2_: O_2 _provides a poor explanation of energy transfer efficiency.

Heat measurements that are independent of carbon dioxide production reveal a strong linear relationship between oxygen uptake and the enthalpy of combustion of many organic compounds [22-24]. The calorimetric to respiratory (CR) ratio is similar for both combustion and respiration at -460 kJ. mol O_2_^-1 ^(± 5%) because enthalpy production per electron equivalent approximates -115 kJ·mol O_2_^-1 ^regardless of the carbon source (a carbon atom has four valences so that four electrons represent -460 kJ·mol O_2_^-1 ^± 5%). In this regard, differences in heat production per unit of oxygen among fat and carbohydrate oxidation are better interpreted by bioenergetic explanations of energy transfer as opposed to gas exchange stoichiometry.

Of the ~36 total ATP re-synthesized by complete glucose oxidation, 2 come from glycolysis (~6% of the total) and 34 come from mitochondrial respiration (~94% of the total). The slight 1.5 kJ increase in heat production per oxygen equivalent when carbohydrate is oxidized compared to fat (at 21.1 kJ vs.19.6 kJ) may be better attributed to the small but requisite energy transfer production of heat and entropy during anaerobic substrate level phosphorylation [25]. The anaerobic 1.5 kJ increase represents ~7% of the total heat production of complete glucose oxidation and is similar to the ~6% anaerobic ATP re-synthesis (2 of 36 ATP); like all energy transfer, glycolytic ATP re-synthesis (phosphorylation) is inefficient.

## Bioenergetics and energy expenditure

Glycolytic phosphorylation and mitochondrial respiration represent separate and distinct acts of energy transfer. Glycolysis and glycogenolysis take place in the cytoplasm of cells, within and around the contractile apparatus of muscles for example. Glycolysis and glycogenolysis require multiple enzymes that catalyze proton and electron transfer. Moreover, glycolytic phosphorylation takes place where the useful energy within glucose and glycogen is converted to ATP. These reactions can be summarized as a series of phosphate transfers, phosphate shifts, isomerizations, dehydrations and aldol cleavages [26]. The inefficiency of glycolytic substrate level ATP re-synthesis is a result of heat and entropy production.

In comparison, the mitochondria are distinct double-membrane cellular organelles; these membranes create an effective compartment that is separated from the cellular cytoplasm. Within these membranes are a collection of further enzymes that continue to strip protons and electrons from substrate. Protons and electrons are subsequently delivered by carriers (e.g., NAD^+^) to the electron transport chain (ETC). Energy transfer in the aerobic re-synthesis of ATP is not directly related to enzymatic glycolytic phosphorylation. Instead, reduction of reduced carriers by oxygen is used to create a gradient of protons. Using the inner membrane as a barrier, protons are pumped to one side; the subsequent gradient of protons creates an uphill-downhill energy transfer scenario whereby specific membrane portals known as mitochondrial ATPases allow protons to pass through. The energy of this downhill flow is exploited to re-synthesize ATP [26]. Mitochondrial heat production has been traced largely to the flow of protons down this gradient [6].

Contemporary bioenergetic interpretations of anaerobic and aerobic metabolism recognize the energy transfer independence of anaerobic and aerobic ATP re-synthesis; each has different reactants and products, uses dissimilar enzymes, involves different types of biochemical reactions, takes place in separate cellular compartments, exploits different types of gradients and, ultimately, each operates with different efficiency [27]. Thus, the heat and entropy production of anaerobic metabolic energy transfer can not possibly be represented by mitochondrial respiration (or vice-versa for that matter). Dissimilar energy transfer formats and operational efficiency must both be kept soundly in mind when interpreting energy expenditure. Nonetheless, glycolytic phosphorylation can proceed *aerobically *whereby pyruvate is immediately and directly routed for mitochondrial respiration (within the Krebs cycle). When the rate of glycolytic phosphorylation (with 2 ATP; 1.5 kJ per l O_2_) matches the rate of mitochondrial respiration (with 34 ATP; 19.6 kJ per l O_2_) then the anaerobic and aerobic components of glucose and glycogen oxidation can be added together to interpret the collective ATP turnover with the energy expenditure conversion, 21.1 kJ per liter of O_2 _(~36 ATP).

## Lactate production

Anaerobic glycolysis and glycogenolysis can proceed by the *rapid *reduction of pyruvate to form lactate (i.e., exceeding mitochondrial respiratory rates and regardless of oxygen availability). In an open system the rate of energy transfer and alterations in the product to reactant ratio can promote greater inefficiency [2,28]. When rapid glycolytic ATP re-synthesis exceeds mitochondrial rates, lactate and heat production ensues and a measure of oxygen uptake no longer accurately reflects the rate or the amount of ATP re-synthesis that takes place. Recall that the calorimetric to respiratory (CR) ratio during respiration is -460 kJ·mol O_2_^-1 ^(± 5%). In cultured mammalian cells however, the ratio of heat production to oxygen uptake was found to vary from -490 to -800 kJ·mol O_2_^-1 ^or more [12]. Gnaiger and Kemp found that the -30 kJ to -340 kJ·mol O_2_^-1 ^increase was best related to the increase in lactate formation and presumably an increase in the anaerobic energy expenditure contribution to total ATP re-synthesis [12,17].

Lactate production in resting fully oxygenated cells is readily apparent [12,16,29,30]. In addition to providing ATP, rapid glycolytic phosphorylation has been suggested to maintain the redox potential within mammalian cells [31], to protect cells against oxidative stress [32], to promote the formation of biosynthetic precursors in growing cells [33] and as a mechanism of control in cellular growth [34]. Whatever its role, rapid glycolytic ATP re-synthesis with lactate production is associated with heat and entropy production and by definition inefficiency and energy expenditure. It appears that the most important step for heat production during rapid rates of glycolysis and glycogenolysis is the reduction of pyruvate to lactate at ^-^63 to ^-^80 kJ per mol of lactate (dependent on the immediate internal and external environments) [12,35]. This energy expenditure is irreversible.

## Lactate removal

Removal of lactate involves conversion back to pyruvate. Pyruvate, in turn, can be converted into a variety of compounds that may include glucose within the liver (Cori Cycle), glycogen within cells (gluconeogenesis) or alanine (an amino acid). It is presumed that the ATP turnover that is required for these conversions comes from mitochondrial energy transfer (as 19.6 kJ per l O_2_) [22].

Lactate can also be removed via the complete aerobic oxidation of pyruvate [36]. The application of energy conservation as expressed in Hess's law (reactions that start and end with the same reactants and products produce the same amount of enthalpy regardless of path) led to the idea that anaerobic energy expenditure during exercise could be measured via subsequent oxygen uptake during the recovery from exercise, as part of the so-called "oxygen debt" [37]. This hypothesis proposes that *all *ATP re-synthesized via glycolytic phosphorylation is included in the net aerobic ATP yield when pyruvate undergoes subsequent aerobic oxidation (36 ATP; 21.1 kJ per l O_2_), even if it passes transiently through lactate. Gaesser and Brooks argued that the *many *fates of lactate and pyruvate removal in addition to complete aerobic oxidation indicate that the oxygen debt does not adequately represent anaerobic glycolytic energy expenditure [38]. Moreover, both aerobic and anaerobic biochemical reactions are often held far-from-equilibrium as part of an open system and this occurs at an irreversible expense [2,18,19].

Strict application of Hess's law to the *in vitro *exothermic reaction of pyruvate to lactate requires that the reverse reaction should consume an equivalent amount of heat. While this is true within closed systems it should not be the case within an *in vivo *open system. It is in the heat loss (calorimetric) to oxygen uptake (respiratory) ratio (kJ·mol O_2_^-1^) that this is most clearly revealed. We found that in cell preparations and cardiac muscle fibers that respire on externally supplied pyruvate or lactate, there is equivalent heat production when expressed per mol of oxygen uptake [20]. That is, heat is not consumed when lactate is converted back to pyruvate; the reaction is not thermodynamically reversible, energy transfer during mitochondrial respiration does not represent energy transfer in the form of rapid or accelerated anaerobic glycolytic ATP re-synthesis with lactate formation. It is therefore ironic that for most of the 20^th ^century muscle cells were known to be chemo-mechanical converters as part of an open system yet energy transfer, as described by the oxygen debt hypothesis, continued to be explained from a traditional thermodynamic closed system standpoint.

## Application and interpretations

**Figure 1:**
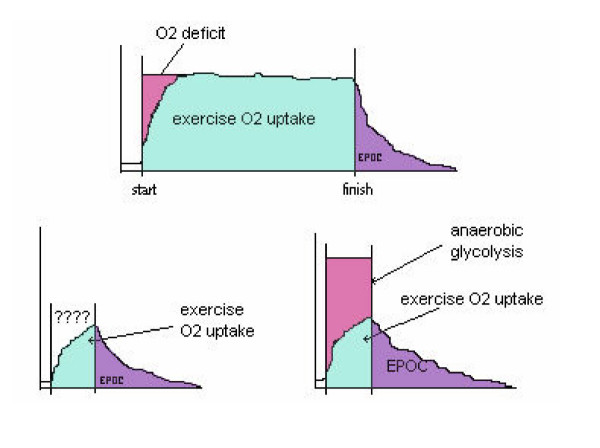
In the top figure, oxygen deficit (pink) represents the anaerobic energy expenditure component to exercise: rapid glycolytic ATP re-synthesis and the use of stored ATP/PC. In this bout of long duration, low to moderate intensity, steady state exercise, the rapid glycolytic component does not make a significant contribution to total energy expenditure. The bottom left figure reveals oxygen uptake measurements for brief, non-steady state, heavy to severe exercise (e.g., a single weight lifting exercise or a quick sprint up a steep hill); vertical lines mark the start and finish to the exercise. The question marks indicate that it is not possible to determine the rapid glycolytic ATP re-synthesis from oxygen-only measurements. The bottom right figure includes a (theoretical) estimate of rapid glycolytic ATP re-synthesis (pink area) and reveals a large absolute and relative anaerobic energy expenditure component to total energy expenditure (restoration of ATP/PC stores are represented in the EPOC measurement).

Indirect calorimetry is a much simpler procedure than direct calorimetry accounting for its continued popularity in estimating biological heat production. When anaerobic energy expenditure contributions are large, however, whole-body energy expenditure may be significantly underestimated (figure 1). It is unfortunate that no valid measure of anaerobic heat production is available – this appears to be another reason for the hesitation to include an anaerobic component as separate from an oxygen-only interpretation of energy expenditure. The problem lies in the inherent difficulties of the collection of anaerobic metabolites from within active cells. Moreover, there are stores of ATP and phosphocreatine (PC) contained within muscle tissue that are utilized during heavy to severe exercise as anaerobic energy transfer but that are re-synthesized aerobically during the recovery from exercise as excess post-exercise oxygen consumption (EPOC). Thus one part of this ATP/ PC turnover is anaerobic, the other is aerobic [38-40]. In fact, heat measurements taken during brief intense exercise have revealed anaerobic metabolism to be more efficient than aerobic metabolism [27]. Such a finding must be considered carefully however as the heat loss during the oxygen deficit portion of exercise contains separate proportions of rapid glycolytic phosphorylation (that represents full ATP turnover) and stored ATP/ PC usage (but not ATP/PC re-synthesis) [40].

There are non-invasive methodologies that estimate only the anaerobic substrate level phosphorylation component of anaerobic energy expenditure (i.e., glycolysis and glycogenolysis without the ATP/ PC stores). One such estimate suggests that every millimole of blood lactate above resting levels equals an energy expenditure of 3 milliliters of oxygen uptake per kilogram of body weight [41]. For example, a 65 kg woman with a resting blood lactate level of 1.1 mmol engages in a 400 meter sprint to exhaustion. Peak lactate levels for her sprint are 12.1 mmol so that the change in blood lactate is 11.0 mmol, resulting in an anaerobic energy expenditure contribution of ~45 kJ (~11 kcals).

Because blood lactate concentrations provide at best an approximate description of muscle lactate levels and glycolytic ATP re-synthesis, it is clear that more research is needed to obtain a valid estimate of anaerobic energy expenditure (concentrations of lactate within active muscle are almost always higher than blood) [42]. On the other hand, the potential error of not including an estimate of anaerobic energy expenditure can result in further misinterpretation [20,43,44]. How important is it to include an estimate of anaerobic energy expenditure for the interpretation of whole-body thermogenesis? Below are a few examples where anaerobic energy expenditure contributions may be sufficiently large that their inclusion may improve current interpretations of whole-body energy expenditure.

## Exercise energy expenditure

It has been concluded from exercise oxygen uptake-only measurements that a one-set circuit weight training regimen consisting of 8 exercises was 15 kcals short of meeting the energy expenditure criteria for a healthy lifestyle in men (i.e., 150-200 kcals per exercise session) [45]. However, these criteria would appear to have been met if an estimate of rapid glycolytic ATP re-synthesis were included with the exercise oxygen uptake measurements. Depending on the size of the exercising muscle mass, my students and I have found blood lactate contributions to a single bout of weight training exercise (i.e., 1 set) to range from 3 to 12 kcalories in men; a minimal contribution of 3 kcal per exercise would result in an increase in energy expenditure of almost 25 kcal for this weight training circuit. The use of both an anaerobic estimate and an aerobic measure of energy expenditure would provide support for regular circuit weight training as an effective method of obtaining a healthy lifestyle in men. The anaerobic energy expenditure component needs to be large to make a significant contribution to total energy expenditure and this is best seen during brief heavy to severe exercise (total energy expenditure includes exercise anaerobic and aerobic energy expenditure and an acute measure of EPOC) (Figure 1).

The effect of anaerobic energy expenditure on total energy expenditure can be seen in the observation that exercise duration and intensity in reptiles and humans have been shown to affect EPOC size [46-48]. It may be inferred that anaerobic and aerobic energy expenditure interact to promote a larger EPOC. In sprinting mice however, EPOC has been found to be independent of either exercise duration or intensity [49]. Mice are very aerobic and may have a limited anaerobic energy expenditure contribution to sprinting, explaining why EPOC volumes are limited in sprinting mice. Unfortunately anaerobic energy expenditure was not estimated in the mouse study. It is of interest to speculate whether, if energy transfer as rapid glycolytic ATP re-synthesis had originally been considered separate from oxygen uptake, the concept of oxygen debt would have been recognized as an interaction between aerobic and anaerobic energy expenditure (metabolism) rather than being interpreted as "repayment on a loan."

## Exercise economy

Exercise economy is traditionally defined as the oxygen uptake required to perform a bout of work at a given rate (e.g., a specific running or cycling pace). During steady state light to moderate intensity exercise, oxygen uptake remains level and provides a sufficient measure of economy. However oxygen uptake steadily increases as heavy to severe steady-state work continues (with ultimate exhaustion) and this has been termed the "slow oxygen uptake component" [50]. This phenomenon remains, for the most part, unexplained yet it is thought that motor unit recruitment patterns may be altered resulting in "additional energy expenditure" [50]. The term "slow oxygen uptake component" implies an aerobic-only approach because the anaerobic glycolytic component is a well known part of heavy to severe exercise. Bioenergetic interpretations might suggest that "additional energy expenditure" is the result of the further dissipation of Gibbs free energy under cellular conditions where both anaerobic and aerobic energy expenditure contributions are changing [2,28].

**Figure 2:**
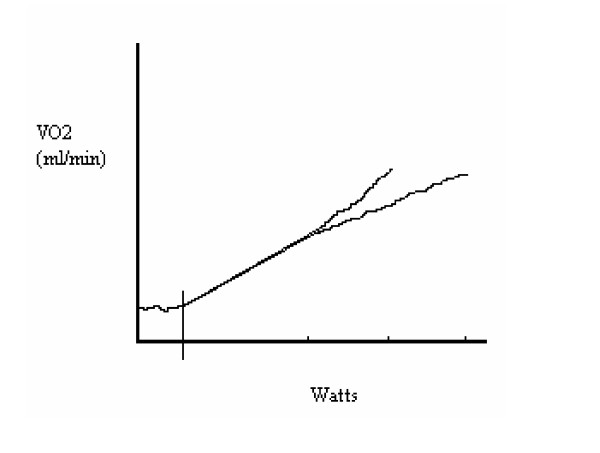
Continuously increasing ramp exercise tests to exhaustion. Resting oxygen uptake is seen until the start of exercise (vertical line). At low to moderate work rates the oxygen uptake to Watts ratio is similar and linear for both slow (e.g., 15 Watts·min^-1^) and fast (e.g., 60 Watts·min^-1^) ramping tests. As the exercise intensity becomes "heavy to severe", the oxygen uptake to Watts ratio increases for the slow ramp test (top line). The opposite is true for the fast ramp test to exhaustion where the oxygen uptake to Watt ratio decreases (bottom line). Notice that the peak Watts are significantly different but the VO_2 _maximum for the two tests is similar [51, 52]. Contributions of both anaerobic and aerobic energy transfer may explain these apparently disparate phenomena as described in the text.

Ramp-type stress tests, unlike steady-state exercise, utilize a continually increasing power output until the test is terminated at exhaustion (figure 2). At low to moderate workloads, oxygen uptake and power output are linear for slow and fast ramp testing, but this is not seen at heavy to severe workloads [51,52]. Slow ramps to exhaustion have gradual increases in power output so that the test can be lengthy, lasting many minutes. Toward the end of a slow ramping test, the ratio of oxygen uptake to power output begins to *increase *so that exercise oxygen uptake appears to contain a "slow oxygen uptake component"; a larger relative aerobic versus anaerobic energy expenditure component is found with slow ramping [52]. On the other hand, fast ramping utilizes rapidly increasing power outputs that promote fatigue quickly, resulting in brief test lengths. Toward the end of a fast ramping test to exhaustion the ratio of oxygen uptake to power output may *decrease*, the traditional interpretation being that this *promotes *larger relative anaerobic energy expenditure. An alternative explanation is that the decrease in the rate of oxygen uptake is *caused *by a faster rate of rapid glycolytic phosphorylation that results in a larger relative anaerobic energy expenditure contribution; that is, a whole-body "Crabtree effect" where a non-linear component to "additional energy expenditure" in the form of anaerobic energy transfer is found [53]. Measures of economy for all types of exercise testing would be improved by an estimate of anaerobic energy expenditure.

## Exothermic to endothermic transition

Mammals are avid consumers of oxygen and well known producers of heat. Mammalian cellular membranes have been shown to leak ions at a rate that is several-fold greater than those in reptiles; the result is an obligatory increase in ion pumping to maintain the electro-chemical membrane potential [54]. Stevens [55] has suggested that stimulation of the sodium pump was an important evolutionary development toward endothermy. Brisk activity of the sodium pump necessitates a rapid rate of ATP re-synthesis. If this is true then it is important to recognize that in some cells lactate with presumed heat production is better correlated with sodium and potassium pumping than is oxygen uptake [29]. The removal of lactate as provided by mitochondrial ATP re-synthesis further contributes to heat production (e.g., Cori cycle, gluconeogenesis, aerobic oxidation). Because resting lactate turnover in endotherms is as much as 1,500-fold higher than in a similar sized ectotherm, the potential for extensive anaerobic ATP re-synthesis needs to be considered as part of basal whole-body thermogenesis in mammals [56]. It seems logical to conclude that most mammalian energy expenditure does come from aerobic metabolism but the evolution of a metabolic acceleration with concomitant heat production comes from both anaerobic and aerobic pathways. The relative contributions of each pathway to whole-body thermogenesis are not known.

## Arousal from torpor

Tucker [11] has shown that heat production in hibernating mice as estimated by oxygen uptake does not account for all of the temperature increases when mice arouse from their metabolic torpor. It is possible therefore that heat production can be accounted for in full when anaerobic energy expenditure is considered as an addition to oxygen-only measurements. Arousal from torpor often induces intense shivering that promotes rapid glycogen degradation accompanied by lactate production and perhaps, like heavy to severe exercise, additional heat production above oxygen uptake-only estimates [57]. The addition of an anaerobic-heat component to whole-body oxygen uptake would appear beneficial to thermogenesis during arousal. Lactate may later be re-converted back to glycogen, a process that may be fueled by mitochondrial fat oxidation to conserve glycogen stores. Such a "'futile cycle" of lactate turnover that is, rapid glycogenolysis (lactate appearance) coupled to gluconeogenesis (lactate disappearance to form glycogen) would be of importance to an obese hibernator who undergoes multiple arousal periods over the course of a winter and has limited access to carbohydrate but has substantial body fat reserves.

## Synopsis

Metabolic energy transfer takes place in part as the oxidation of carbohydrate that includes an anaerobic (glycolysis) and aerobic (mitochondrial) component. Rapid glycolytic ATP re-synthesis with lactate production can exceed mitochondrial rates and under these conditions the efficiency of anaerobic energy transfer can not be interpreted using gas exchange stoichiometry. When rapid glycolytic ATP re-synthesis with concomitant heat production is extensive, the anaerobic contribution to energy expenditure can be significant both in cells and in whole-animals. The interpretation of efficiency and energy expenditure may be improved if a separate estimate of anaerobic ATP turnover is provided along with a measure of oxygen uptake.
